# Salvianolic Acid B improves cognitive impairment by inhibiting neuroinflammation and decreasing Aβ level in *Porphyromonas gingivalis*-infected mice

**DOI:** 10.18632/aging.103306

**Published:** 2020-06-09

**Authors:** Jianwei Liu, Yiling Wang, Jing Guo, Jinyan Sun, Qinfeng Sun

**Affiliations:** 1 Department of Orthodontics, Jinan Stomatological Hospital, Jinan, China; 2 Department of Orthodontics, School and Hospital of Stomatology, Cheeloo College of Medicine, Shandong University and Shandong Key Laboratory of Oral Tissue Regeneration and Shandong Engineering Laboratory for Dental Materials and Oral Tissue Regeneration, Jinan, Shandong Province, China; 3 School of Stomatology, Qingdao University, Qingdao, Shandong Province, China

**Keywords:** Salvianolic Acid B, *Porphyromonas gingivalis*, neuroinflammation, Aβ metabolism

## Abstract

Amyloid-β (Aβ) accumulation is one of the main pathological hallmarks of Alzheimer’s disease (AD). *Porphyromonas gingivalis* (*P. gingivalis*), the pathogen of chronic periodontitis, could cause Aβ accumulation and was identified in the brain of AD patients. Salvianolic Acid B (SalB) has been proven to have the neuroprotective effect. Whether SalB could protect against *P. gingivalis*-induced cognitive impairment is still unknown. In this study, a *P. gingivalis*-infected mouse model was employed to study the neuroprotective role of SalB. The results showed that SalB (20 and 40 mg/kg) treatment for 4 weeks could shorten the escape latency and improve the percentage of spontaneous alternation in the *P. gingivalis*-infected mice. SalB inhibited the levels of reactive oxygen species and malondialdehyde, while increased the levels of antioxidative enzymes (superoxide dismutase and glutathione peroxidase). SalB decreased the levels of IL-1β and IL-6, increased the mRNA levels of *bdnf* and *ngf* in the brain of *P. gingivalis*-infected mice. In addition, SalB obviously decreased the level of Aβ. SalB elevated the protein expression of ADAM10, while downregulated BACE1 and PS1. SalB increased the protein expression of LRP1, while decreased RAGE. In conclusion, SalB could improve cognitive impairment by inhibiting neuroinflammation and decreasing Aβ level in *P. gingivalis*-infected mice.

## INTRODUCTION

Alzheimer’s disease (AD) is an age-related chronic neurodegenerative disease. In clinical, AD is characterized by gradual decline in memory and cognitive ability [1]. Excessive accumulation of amyloid-β (Aβ) and abnormally hyperphosphorylated tau protein are the main pathologies of AD [2]. The Aβ hypothesis is considered as one of the main reason for AD. Aβ accumulation induces neural oxidative stress, neuroinflammation and apoptosis, which lead to synaptic injury, neuronal death and cognitive impairment [3]. Chronic periodontitis (CP), which is caused by *Porphyromonas gingivalis* (*P. gingivalis*), has been identified as a risk factor for AD [4, 5]. Recently, in AD patients, infectious agents have been found in the brain, which cause neuroinflammation [6, 7]. Aβ seems to be an antimicrobial peptide against infectious microorganisms [8]. *P. gingivalis* lipopolysaccharide (LPS) has been found in AD patients’ brains [9]. Treatments targeting Aβ are considered to slow the progression of AD. However, all of the clinical trials were reported failed [10]. The main reason is that drug treatment is too late during AD course. Currently, there are no drugs that could prevent or delay AD progression. Thus, finding effective therapeutic drugs targeting *P. gingivalis* is a possible way for AD.

**Figure 1:**
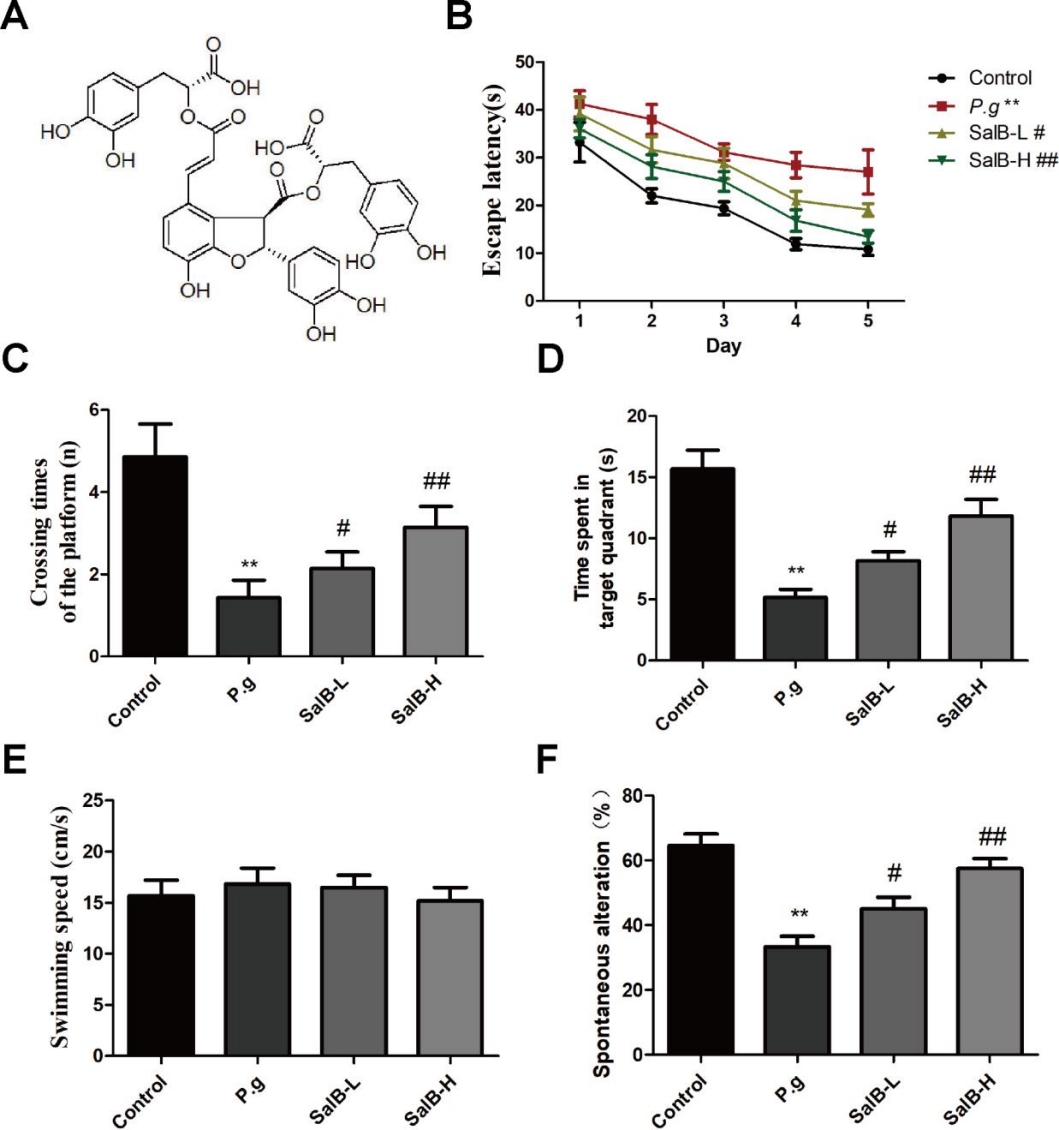
**SalB improves memory deficits in *P. gingivalis*-infected mice.** (**A**) The molecular structure of SalB; (**B**) Escape latency of Morris Water Maze test; (**C**) Crossing times of the platform; (**D**) Time spend in the target quadrant; (**E**) Swimming speed; (**F**) Percentage of spontaneous alternation of Y-maze. Experimental values were expressed as mean ± SEM (n = 15 per group). *P < 0.05, **P < 0.01 vs. Control; ^#^*P* < 0.05, ^##^*P* < 0.01 vs. *P.g*.

*Salvia miltiorrhiza* is a common Chinese medicinal herb in China. It contains some potential active ingredients, including Salvianolic acid B (SalB; Figure 1A). SalB is extracted from the roots of *Salvia miltiorrhiza* *Bunge* [11]. SalB have some well-known pharmacological actions, such as anti-oxidative stress, anti-inflammation [12–14]. Previous studies have indicated that SalB could protect against cardiovascular diseases [15–17]. In addition, SalB also possesses neuroprotective effect against AD. SalB can inhibit Aβ aggregation and fibril formation [18]. Besides, SalB can decrease Aβ level by inhibiting BACE1 activity [19, 20]. However, whether SalB could improve the cognitive impairment in *P. gingivalis*-infected mice is unclear.

In this study, a new AD model, *P. gingivalis*-infected mouse model, was employed to investigate the neuroprotective role of SalB. *P. gingivalis* infection can cause Aβ aggregation and neuroinflammation in the brain, which is similar to AD pathology. Different doses (20 and 40 mg/kg) of SalB were used to treat the mice. We revealed that SalB could obviously protect against *P. gingivalis*-induced memory deficits by inhibiting neuroinflammation and decreasing Aβ level.

## RESULTS

### SalB improves memory deficits in *P. gingivalis*-infected mice

In order to know whether SalB could improve the cognitive impairment in *P. gingivalis*-infected mice, we firstly tested the effect of SalB on cognitive deficits in *P. gingivalis*-infected mice by behavioral tests. As shown in the Morris water maze test (Supplementary Figure 1, Figure 1B–1E), the escape latency was obviously increased in *P. gingivalis*-infected mice, when compared with control group. However, SalB (20 and 40 mg/kg) significantly shortened the time spent in finding the platform. On the 7^th^ day, we removed the platform to estimate the spatial working memory. The *P. gingivalis*-infected mice had less crossing times and spent shorter time in the target quadrant than control group. The SalB groups increased crossing times and shortened time spent in the target quadrant. The average swimming speed was similar among all groups (Figure 1E). The Y-maze spontaneous alternation test was also used to detect the neuroprotective effect of SalB (Figure 1F). The spontaneous alternation index was impaired in *P. gingivalis*-infected mice. SalB could improve the spontaneous alternation in *P. gingivalis*-infected mice. The data suggested that SalB had neuroprotective effect against memory impairment in *P. gingivalis*-infected mice.

### SalB ameliorates oxidative stress and neuroinflammation in *P. gingivalis*-infected mice

**Figure 2:**
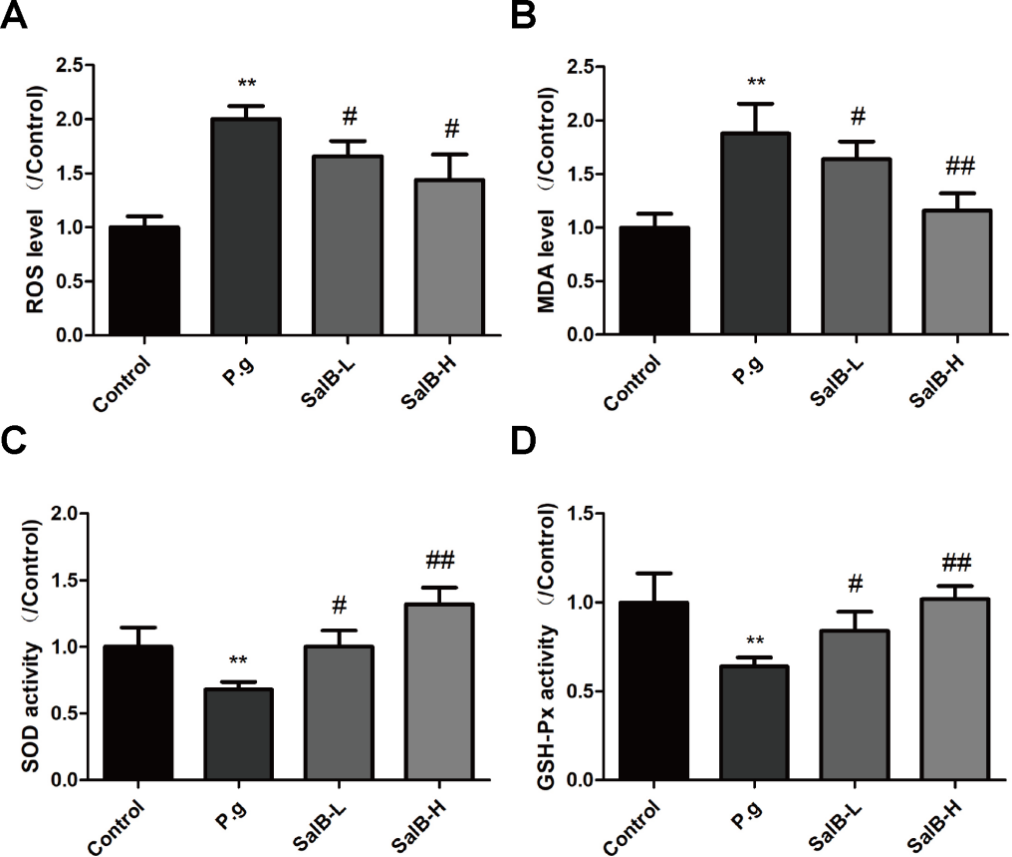
**SalB ameliorates oxidative stress in *P. gingivalis*-infected mice.** The levels of ROS (**A**), MDA (**B**) and the enzymes activities of SOD (**C**), GSH-Px (**D**) were detected in the hippocampus of *P. gingivalis*-infected mice. Experimental values were expressed as mean ± SEM (n = 6 per group). *P < 0.05, **P < 0.01 vs. Control; ^#^*P* < 0.05, ^##^*P* < 0.01 vs. *P.g.*

**Figure 3:**
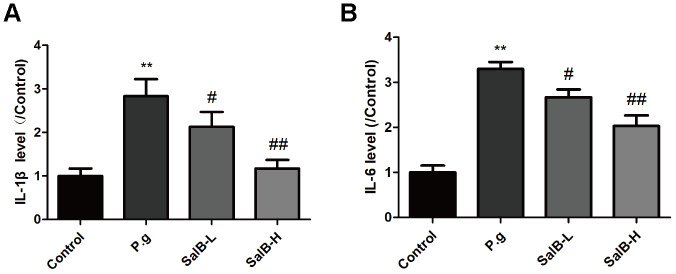
**SalB ameliorates neuroinflammation in *P. gingivalis*-infected mice.** The inflammatory factors levels of IL-1β (**A**) and IL-6 (**B**) in the hippocampus of *P. gingivalis*-infected mice. Experimental values were expressed as mean ± SEM (n = 6 per group). *P < 0.05, **P < 0.01 vs. Control; ^#^*P* < 0.05, ^##^*P* < 0.01 vs. *P.g.*

We further investigated the protection against oxidative stress and anti-neuroinflammation effects of SalB. As shown in Figure 2, increased levels of oxidative stress indicators, ROS and MDA, as well as suppressed activities of antioxidant enzymes, SOD and GSH-Px, were observed in the hippocampus of *P. gingivalis*-infected mice. SalB significantly decreased the levels of ROS and MDA. Moreover, SalB significantly increased the activities of SOD and GSH-Px. In addition, inflammatory factors (IL-1β and IL-6) were also elevated in the hippocampus of *P. gingivalis*-infected mice, compared with control group (Figure 3). SalB significantly decreased the levels of IL-1β and IL-6. These results indicated that the neuroprotective effects of SalB could be related to its protection against oxidative stress and anti-neuroinflammation effects.

### SalB improves the expressions of neurotrophic factors in *P. gingivalis*-infected mice

**Figure 4:**
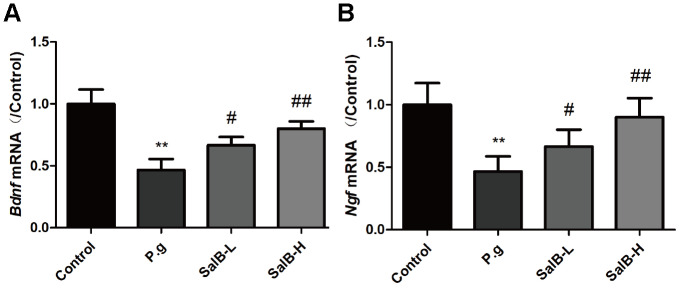
**SalB improves neurotrophic factors in *P. gingivalis*-infected mice.** The mRNA levels of *bdnf* (**A**) and *ngf* (**B**) were detected in the hippocampus of *P. gingivalis*-infected mice. Experimental values were expressed as mean ± SEM (n = 6 per group). *P < 0.05, **P < 0.01 vs. Control; ^#^*P* < 0.05, ^##^*P* < 0.01 vs. *P.g*

We also tested the neurotrophic factors (BDNF and NGF) to evaluate the nervous system function of *P. gingivalis*-infected mice. As shown in Figure 4, the mRNA levels of *bdnf* and *ngf* were decreased in the hippocampus of *P. gingivalis*-infected mice. After treatment with SalB, the mRNA levels of *bdnf* and *ngf* were increased. These data suggested that SalB could increase the gene expressions of *bdnf* and *ngf*.

### SalB decreases Aβ metabolism in *P. gingivalis*-infected mice

**Figure 5:**
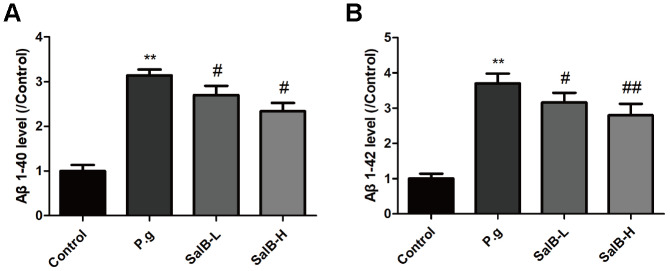
**SalB decreases Aβ levels in *P. gingivalis*-infected mice.** The levels of Aβ1-40 (**A**) and Aβ1-42 (**B**) were detected in the hippocampus of *P. gingivalis*-infected mice. Experimental values were expressed as mean ± SEM (n = 6 per group). *P < 0.05, **P < 0.01 vs. Control; ^#^*P* < 0.05, ^##^*P* < 0.01 vs. *P.g.*

**Figure 6:**
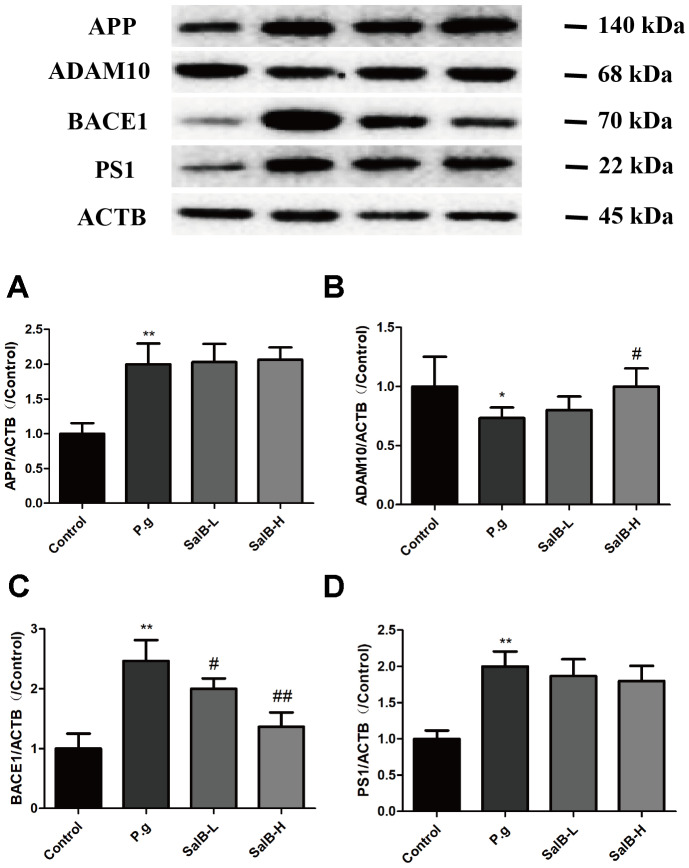
**SalB inhibits Aβ generation in *P. gingivalis*-infected mice.** Western blot results of APP (**A**), ADAM10 (**B**), BACE1 (**C**) and PS1 (**D**) were detected in the hippocampus of *P. gingivalis*-infected mice. Experimental values were expressed as mean ± SEM (n = 3 per group). *P < 0.05, **P < 0.01 vs. Control; ^#^*P* < 0.05, ^##^*P* < 0.01 vs. *P.g.*

**Figure 7:**
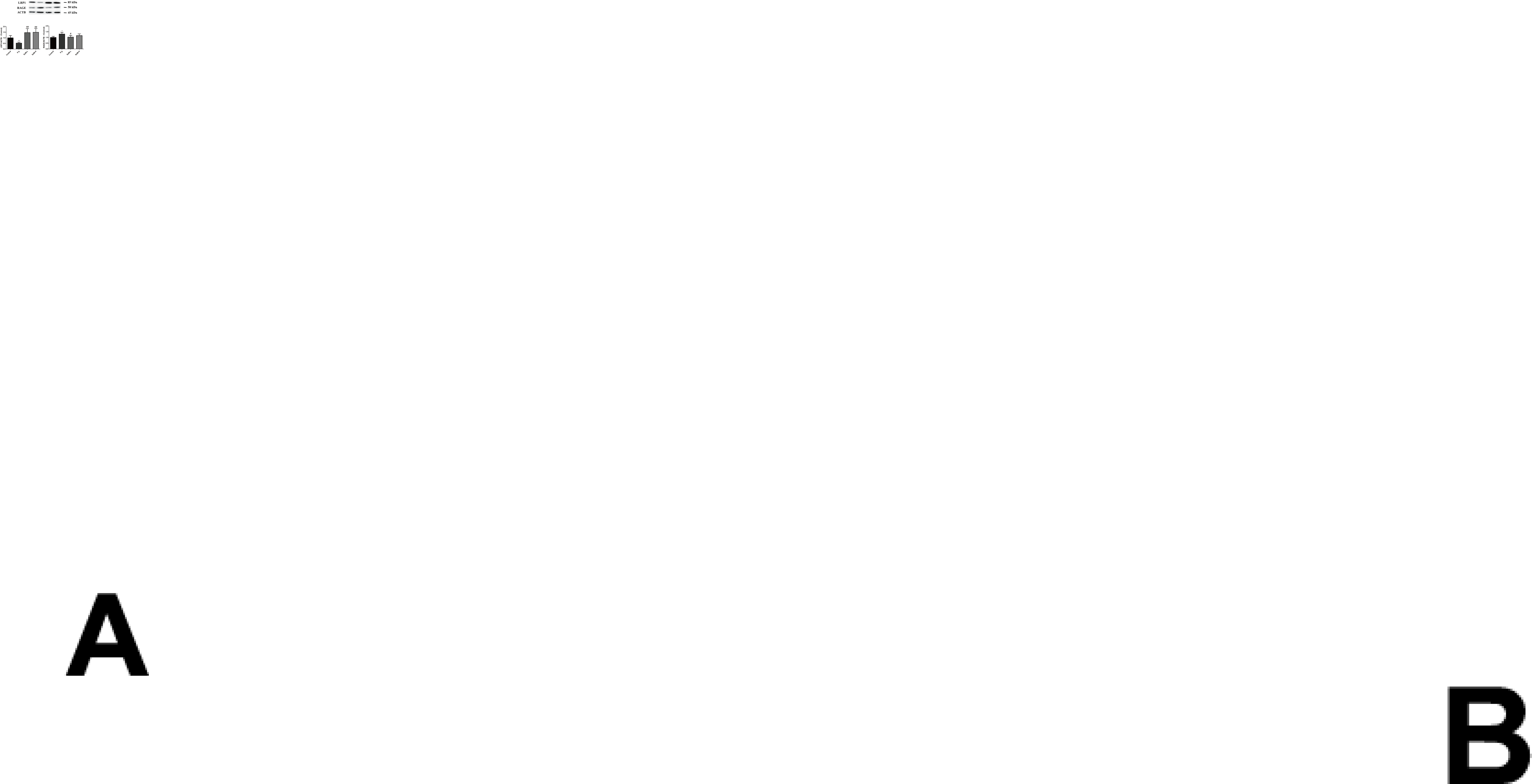
**SalB improves Aβ transportation in *P. gingivalis*-infected mice.** Western blot results of LRP1 (**A**) and RAGE (**B**) were detected in the hippocampus of *P. gingivalis*-infected mice. Experimental values were expressed as mean ± SEM (n = 3 per group). *P < 0.05, **P < 0.01 vs. Control; ^#^*P* < 0.05, ^##^*P* < 0.01 vs. *P.g.*

In order to illustrate whether the neuroprotective effect of SalB was related to Aβ, we tested the Aβ level in the hippocampus of *P. gingivalis*-infected mice (Figure 5). Both Aβ 1-40 and Aβ 1-42 were obviously increased in the hippocampus of of *P. gingivalis*-infected mice. SalB effectively decreased the Aβ 1-40 and Aβ 1-42 levels. We further studied the related mechanisms. As shown in Figure 6, SalB could elevate the protein expression of ADAM10, while downregulate the protein expressions of BACE1 and PS1. These data indicated that SalB could inhibit Aβ generation, which was in accord with previous study. In addition, Aβ transportation-related proteins were also detected. SalB increased the protein expression of LRP1 and decreased the protein expression of RAGE (Figure 7). These data suggested that Aβ generation and transportation mechanisms were also involved in the neuroprotective effects of SalB.

## DISCUSSION

In this study, we used a new AD animal model, *P. gingivalis*-infected mouse model, to study the neuroprotective effect of SalB. *P. gingivalis* can be found AD patients’ brains and cause Aβ aggregation and neuroinflammation. Behavioral tests (Morris Water Maze test and Y-maze test) suggested that SalB (20 and 40 mg/kg) could ameliorate the memory impairment in *P. gingivalis*-infected mice. Moreover, SalB could protect neuron against oxidative stress and increase the mRNA levels of neurotrophic factors. In addition, SalB could ameliorate neuroinflammation, inhibit Aβ generation and promote Aβ transportation.

*P. gingivalis* is mainly found in gingival and periodontal infections [21]. Recent studies have proven that patients with *P. gingivalis* infection could develop into AD or hasten the progression of AD [6, 22]. Periodontitis allows the bacteria entering the whole body through the bloodstream. AD is clinically characterized by the impairment of learning and memory. A great deal of studies showed that Aβ plays the central role in AD development [23–25]. *P. gingivalis* can cause Aβ accumulation, which initiates the cascade reaction in AD process. In this study, *P. gingivalis*-infected mice were employed to mimic AD pathology. To study the neuroprotective effect of SalB, two behavioral tests, including Morris water maze and Y-maze tests, were used to evaluate the learning and memory ability. Results indicated that SalB treatment protected against memory impairment in the *P. gingivalis*-infected mice.

SalB, an active compound from the roots of *Salvia miltiorrhiza* *Bunge*, has been revealed to neuroprotective effects [26, 27], which is mainly related to the anti-Aβ fiber and antioxidant effects [18, 28, 29]. Aβ accumulation in the brain can induce a series of pathological changes, including oxidative stress. Oxidative stress can cause apoptosis of neuron and induce dysfunction of nervous system [30]. ROS generation and increased level of MDA in the brain are the most important indicators of oxidative stress [31, 32]. In addition, antioxidant enzymes, such as SOD and GSH-Px, can be impaired by Aβ fiber [33]. Consistently, an increased oxidative stress statement and a decrease of neurotrophic factors were observed in the brain of *P. gingivalis*-infected mice. As expected, SalB ameliorated the Aβ-induced oxidative stress and increased the levels of neurotrophic factors in the brain of *P. gingivalis*-infected mice. These findings suggested that of the antioxidant capacity of SalB might be involved in the neuroprotective effect.

Inhibiting Aβ generation is an effective method to prevent Aβ accumulation. Aβ peptides are generated by APP excision. APP is a transmembrane protein, which can be cleaved by α- (ADAM10), β- (BACE1) and γ-secretase (PS1). The cleavage site of ADAM10 can prevent Aβ generation [34, 35]. When APP is mainly cleaved by BACE1, Aβ generation is increased. Thus, activation of ADAM10 and inhibition of BACE1 is an effective path to reduce Aβ accumulation. Previous studies have reported that SalB could inhibit BACE1 activity *in vitro* [19, 20]. In this study, we further studied this *in vivo*. Consistently, SalB decreased the level of Aβ. SalB decreased the protein expressions of BACE1 and PS1 and increased the protein expression of ADAM10 in the brain of *P. gingivalis*-infected mice. Aβ can also be transported across the BBB to the outside of brain. LRP1 is the main transporter for Aβ efflux at the BBB, while RAGE can transport Aβ from the circulation into the brain [36–38]. Thus, activating LRP1 and inhibiting RAGE can prevent Aβ enter the brain. In this study, SalB effectively increased the protein expression of LRP1 and decreased the protein expression of RAGE in the brain of *P. gingivalis*-infected mice. These results indicated that SalB could improve cognitive impairment via regulating Aβ metabolism.

In summary, this study provided some evidences that SalB could improve cognitive impairment via regulating neuroinflammation and Aβ metabolism in *P. gingivalis*-infected mice. SalB might be developed as an anti-AD drug. However, further studies are still needed. We will further study the mechanisms of *P. gingivalis*-induced Aβ accumulation. In addition, the key molecule of the target in SalB’s effect is still needed to identify.

## MATERIALS AND METHODS

### Materials

Salvianolic acid B (SalB, purity > 99%) was purchased from the Chinese National Institute for the Control of Pharmaceutical and Biological Products (Beijing, China). 2’,7’-dichlo-rofluorescin diacetate (DCFH-DA) were obtained from Invitrogen (Carlsbad, CA, USA). Chemical kits used for the detection of malondialdehyde (MDA), superoxide dismutase (SOD), and glutathione peroxidase (GSH-Px) were purchased from the Nanjing Jiancheng Bioengineering Institute (Nanjing, China). The RNeasy kit was purchased from Qiagen (Hilden, Germany). SuperScript III Reverse Transcriptase was purchased from Invitrogen (Carlsbad, CA, USA). ELISA kits used for the detection of IL-1β, IL-6, Aβ1-40 and Aβ1-42 were purchased from Invitrogen. The blots were probed with the following antibodies: anti-Low density lipoprotein receptor-related protein 1 (LRP1, Abcam); anti-receptor for advanced glycation endproducts (RAGE, Abcam); anti-ACTB (Sigma, Aldrich); secondary antibody horseradish peroxidase- (HRP-) conjugated goat anti-rabbit IgG (Cell Signaling Technology). The Western blot chemiluminescent horseradish peroxidase substrate was purchased from Millipore (USA). All other reagents and chemicals used in the study were of analytical grade.

### Animal and treatment

Twelve-month-old male C57BL/6J mice were obtained from the Model Research Centre of Shandong University and were kept in a specific-pathogen-free environment according to protocols approved by the Animal Care and Use Committee of Shandong University. The mice were kept under controlled temperature (22 ± 2 °C) and humidity (55 - 70 %) conditions. A 12-hour light/12- hour dark cycle was maintained with food and water available ad libitum. The mice were kept in the cage for one week to adapt to the environment, a week prior to the experiment.

These mice were infected with *P. gingivalis* according to previous method [22]. Briefly, mice were infected with *P. gingivalis* (1×10^8^ CFU/mouse) every 3 days by intraperitoneally injection for 3 weeks. The mice were randomly assigned to four treatment groups (n = 15 per group): vehicle control group (0.9 % saline), *P. gingivalis*-infected group (P.g), low-dose SalB group (*P. gingivalis*-infected mice, SalB 20 mg/kg/d), high-dose SalB group (*P. gingivalis*-infected mice, SalB 40 mg/kg/d). Mice were treated with saline and SalB, respectively, by gavage, once per day for four weeks.

### Morris water maze test

The Morris water maze test was used to detect spatial learning and memory ability. The procedure that was followed has been mentioned in previous studies [39]. In brief, the mice were subjected to the navigation test for five consecutive days. For each daily trial, there were four sequential training trials. The escape latency was recorded.

### Y-maze test

The Y-maze test was used to evaluate the working memory performance. The procedure has been reported previously [40]. Initially, the mice were placed in one arm of the maze. Subsequently, the number of time each mouse entered the other arm in a 5 min interval was recorded. After behavior tests, all mice were anesthetized with chloral hydrate (intraperitoneal injection) and sacrificed by cervical dislocation. Then, the hippocampus tissues of brain were separated on the ice and frozen in the -80 °C refrigerator for further tests.

### Reactive oxygen species (ROS) level

The tissues of the mice hippocampus were homogenized in cold saline. The ROS level was measured by the DCFH-DA fluorescent dye method. Measure fluorescence (Ex/Em = 485/535 nm) in a microplate reader with a fluorescence microscope. The intracellular ROS level obtained was noted.

### Malondialdehyde (MDA) level, superoxide dismutase (SOD) and glutathione peroxidase (GSH-Px) activities

The tissues of the mice hippocampus were homogenized in cold saline. The homogenate was centrifuged, and the supernatant was collected to detect the level of MDA and the activities of SOD and GSH-Px using kits (Nianjing Jiancheng Bioengineering Institute, Nanjing, China). The procedures mentioned in the manufacturer’s instructions were followed.

### Quantitative PCR

The tissues of the mice hippocampus were homogenized. The total RNA was isolated using an RNeasy kit and reverse-transcribed using SuperScript III Reverse Transcriptase. The gene expression was determined relative to a calibrator and normalized to the housekeeping gene β-actin using the standard curve method. Forward and reverse primers were as follows: *Nerve growth factor* (*Ngf*): For, 5′-CAAGGACGCAG CTTTCTATACTG-3′, Rev, 5′-CTTCAGGGACAGAG TCTCCTTCT-3′; *Brain-derived neurotrophic factor* (*Bdnf*): For, 5′-TACTTCGGTTGCATGAAGGCG-3′, Rev, 5′-GTCAGACCTCTCGAACCTGCC-3′; *β-actin*: For, 5′-AGAGCTACGAGCTGCCTGAC-3′, Rev, 5′-AGCACTGTGTTGGCGTACAG-3′.

### ELISA

The tissues of the mice hippocampus were homogenized in cold saline. The homogenate was centrifuged, and the supernatant was collected to detect the levels of IL-1β, IL-6, Aβ1-40 and Aβ1-42 by using an ELISA kit (Invitrogen) according to the manufacturer’s instruction.

### Western blot analysis

The tissues of the mice hippocampus were homogenized and lysed in the sample buffer. The protein was subjected to SDS-polyacrylamide gel electrophoresis (PAGE) analysis and transferred to PVDF membranes. The membranes were incubated with anti-LRP1, anti-RAGE, anti-ACTB. Then the membrane was incubated with horseradish peroxidase-conjugated anti-rabbit. Finally, the bands on the membrane were visualized.

### Statistical analysis

Analysis of the data was performed using GraphPad Prism (V6, California, USA). The results were expressed as the mean ± SD. All statistical analyses were evaluated using Student’s *t*-test and ANOVA in SPSS 19.0 statistical software (IBM, Endicott, NY). The differences were considered as statistically significant at *p* < 0.05.

## Supplementary Material

Supplementary Figure 1
