# Supplementary material for: Salvianolic Acid B improves cognitive impairment by inhibiting neuroinflammation and decreasing Aβ level in Porphyromonas gingivalis-infected mice
Source: Aging (Albany NY). 2020 Jun 9;12(11):10117–28. doi: 10.18632/aging.103306 (PMC7346047; doi:10.18632/aging.103306)
Supplement: Supplementary Figure 1 [file aging-12-103306-s001..pdf]

SUPPLEMENTARY FIGURE

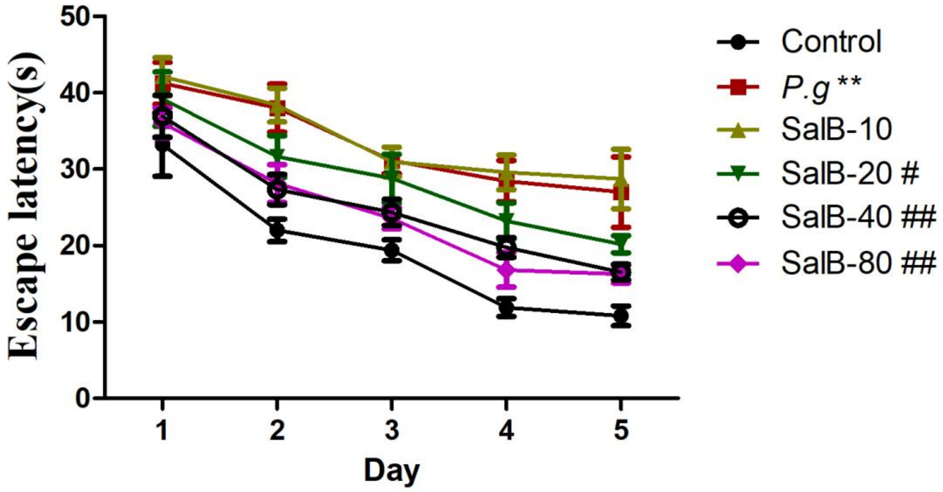

Supplementary Figure 1. Different dosages of SalB protects against memory deficits in *P. gingivalis*-infected mice. Escape latency of Morris water maze test. Experimental values were expressed as mean ± SEM (n = 15 per group). ** P < 0.01 vs. Control; #P < 0.05, ##P < 0.01 vs. *P.g*.
